# Microstructural Correlates of Emotional Attribution Impairment in Non-Demented Patients with Amyotrophic Lateral Sclerosis

**DOI:** 10.1371/journal.pone.0161034

**Published:** 2016-08-11

**Authors:** Chiara Crespi, Chiara Cerami, Alessandra Dodich, Nicola Canessa, Sandro Iannaccone, Massimo Corbo, Christian Lunetta, Andrea Falini, Stefano F. Cappa

**Affiliations:** 1 Università Vita-Salute San Raffaele, Milano, Italy; 2 Division of Neuroscience, San Raffaele Scientific Institute, Milano, Italy; 3 Department of Clinical Neurosciences, IRCCS San Raffaele Turro, Milano, Italy; 4 NeTS Center, Scuola Universitaria Superiore IUSS, Pavia, Italy; 5 Department of Neurorehabilitation Sciences, Casa Cura Policlinico, Milano, Italy; 6 NEuroMuscolar Omnicentre, Fondazione Serena Onlus, Niguarda Ca’ Granda Hospital, Milano, Italy; 7 CERMAC – Neuroradiology, San Raffaele Scientific Institute, Milano, Italy; Duke-NUS Graduate Medical School, SINGAPORE

## Abstract

Impairments in the ability to recognize and attribute emotional states to others have been described in amyotrophic lateral sclerosis patients and linked to the dysfunction of key nodes of the emotional empathy network. Microstructural correlates of such disorders are still unexplored. We investigated the white-matter substrates of emotional attribution deficits in a sample of amyotrophic lateral sclerosis patients without cognitive decline. Thirteen individuals with either probable or definite amyotrophic lateral sclerosis and 14 healthy controls were enrolled in a Diffusion Tensor Imaging study and administered the Story-based Empathy Task, assessing the ability to attribute mental states to others (i.e., Intention and Emotion attribution conditions). As already reported, a significant global reduction of empathic skills, mainly driven by a failure in Emotion Attribution condition, was found in amyotrophic lateral sclerosis patients compared to healthy subjects. The severity of this deficit was significantly correlated with fractional anisotropy along the forceps minor, genu of corpus callosum, right uncinate and inferior fronto-occipital fasciculi. The involvement of frontal commissural fiber tracts and right ventral associative fronto-limbic pathways is the microstructural hallmark of the impairment of high-order processing of socio-emotional stimuli in amyotrophic lateral sclerosis. These results support the notion of the neurofunctional and neuroanatomical continuum between amyotrophic lateral sclerosis and frontotemporal dementia.

## Introduction

Amyotrophic Lateral Sclerosis (ALS) is a heterogeneous multi-component disease, including in about 50% of cases non-motor dysfunctions encompassing the cognitive and/or behavioral changes typically observed in the behavioral variant of frontotemporal dementia (bvFTD) [1]. More specifically, such extra-motor impairments can range from the identification of isolate deficits to the fulfillment of the criteria for the diagnosis of frontotemporal dementia (FTD), and particularly of the bvFTD syndrome [2–3]. The latter condition affects about 10–15% of ALS cases [4–6]. Notably, an overlap between the two conditions has also been observed at genetic and neuropathological levels, supporting the ALS-FTD continuum hypothesis [7–10]. Neuroimaging findings provided further suggestive evidence, by showing the pathological involvement of frontal and temporo-limbic regions in ALS patients [11–13].

Cognitive extra-motor impairments in ALS may involve language, memory and executive abilities [4, 6, 14], while the major behavioral symptom is apathy [15–17]. Many studies also underlined a set of alterations specifically related to the failure of social cognition skills, i.e. one of the core features of the bvFTD syndrome [3], and social cognition deficit is now recognized as one of the main cognitive signatures possibly occurring in ALS [18]. In particular, social cognition impairments include a constellation of dysfunctions ranging from the simple processing of socio-emotional stimuli–e.g., identification of basic emotions from facial expression [19–20], social judgments and memory for emotional material [21–23], affective decision-making [24–25]–to more complex functions such as empathy [26–28], a psychological construct reflecting the overall ability to attribute mental states to others in order to understand their behavior in the social context.

Empathy is usually broken down into two main components [29]. The first one relies on the production of a visceromotor representation of affective states of others, allowing the experience of their feelings (i.e., emotional contagion or *emotional empathy*). The second aspect is the computation of others’ cognitive states and intentions (i.e., perspective-taking/mentalizing skills or *cognitive empathy*). At the neural level, while the cognitive facet of empathy engages a temporo-parietal network (i.e., superior temporal sulcus, temporo-parietal junction, medial prefrontal cortex), emotional attribution abilities require a distinct system, involving a set of fronto-limbic regions (i.e., inferior frontal gyrus, anterior insula, anterior cingulate cortex) [30–31].

Given the early and progressive degeneration of frontal and limbic structures in bvFTD patients [32–33], the impairment of components of social cognition skills in these subjects is not unexpected [34–37], and indeed represents a key feature of the disease [3]. There is now a growing evidence of deficits in the recognition and processing of socio-emotional information in ALS [18], compatible with an overlap between ALS and FTD. Indeed, such impairments show common structural and functional underpinnings in the two clinical syndromes [18, 38]. Impaired performance in a task assessing emotional attribution have been reported in both ALS and bvFTD [27, 36, 39]. This was related to macrostructural changes in fronto-limbic structures representing key nodes of the neural system supporting emotional empathy [27, 36]. To date, no study specifically explored the microstructural white-matter correlates of the processing of emotional stimuli in ALS. The assessment of brain connectivity within the fronto-limbic circuitry with the Diffusion Tensor Imaging (DTI) techniques may offer useful suggestions about the impairment of specific social cognition networks in ALS and increase our comprehension of the pathological ALS-FTD continuum.

## Materials and Methods

### Participants

Thirteen subjects with a diagnosis of either probable or definite ALS [40] who did not have dementia (10 males and 3 females, mean age = 58.97±10.57 years; mean education = 11.5±4.41 years), and 14 age-, gender- and education-matched healthy controls (HC) were recruited in the DTI study. The patients were a subsample of the group reported by Cerami and colleagues [27]. See Table 1 for demographic and clinical details.

**Table 1:** Demographic information and clinical characteristics.

	ALS (n = 13)	HC (n = 14)	p-value
Gender (male: female)	10: 3	9: 5	0.47
Mean age (years)	58.97 ± 10.57	56.12 ± 7.79	0.42
Mean education (years)	11.5 ± 4.41	14 ± 3.66	0.12
Disease Duration (months from symptoms onset)	25 ± 22.37	-	-
ALS-FRSr	39.38 ± 5.91	-	-

The table shows the proportion of males/females, mean values and standard deviations corresponding to age and education (in all groups), and clinical variables (in patients). P-values relative to the comparison between patients and controls are also provided. ALS, Amyotrophic Lateral Sclerosis; HC, Healthy Controls.

ALS patients underwent a structured clinical interview, a full neurological examination, and a Magnetic Resonance Imaging (MRI) investigation, including T1, T2, and FLAIR sequences, performed for diagnostic purposes. All ALS cases had no known family history. Patients were routinely screened for pathogenic mutations on *C9ORF72* and *GRN* genes. No patient carried known mutations. Exclusion criteria were left-handedness, a positive history for other neurological or psychiatric disorders, and the presence of other pathological evidence on MRI scan. We also excluded patients with respiratory disorders (forced vital capacity <70% of predicted capacity), severe dysarthria and communication difficulties potentially invalidating the interpretation of neuropsychological performances. According to the disease-onset type, 3 patients out of 13 had a bulbar-onset disease (i.e., dysarthria and dysphagia), while the rest presented with spinal-onset. Patients’ disability was evaluated with the revised version of the ALS-Functional Rating Scale (ALSFRS-r) [41].

All ALS subjects completed a standard neuropsychological evaluation to assess the presence of cognitive and/or behavioral impairments. Specifically, we assessed language (picture naming and single word comprehension), memory (verbal working-memory: digit span forward; long-term memory: Rey Auditory Verbal Learning test), and executive functions (Raven Colored Progressive Matrices; digit span backward; letter and category fluency tests; Cognitive Estimation Task; Stroop interference test and either Wisconsin Card Sorting Test or Weigl’s Sorting Test), as well as the presence of behavioral dysfunctions (Frontal Behavioral Inventory and Neuropsychiatric Inventory). On the basis of the presence/absence of cognitive and/or behavioral impairments, and according to Strong’s consensus criteria [42], we identified 2 ALS patients with dysexecutive deficits (i.e., ALSci) and 2 additional patients with behavioral disorders (i.e., apathy, irritability and disinhibition, namely ALSbi). No patient with combined ALSci/bi syndrome was identified. The remaining patients (i.e., 9/13, 70%) were cognitively and behaviorally unimpaired (i.e., pure ALS).

Cognitively normal subjects (HC) were recruited from local senior community centers. Inclusion criteria were the absence of neuropsychiatric disorders, negative neurologic examination, global Clinical Dementia Rating score = 0, Mini-Mental State Examination score ≥28/30, verbal and visuo-spatial delayed memory performance (Rey Auditory Verbal Learning test and Rey Figure Recall task) ≥25^th^ percentile. None of the HC was taking any medication potentially interfering with neurobehavioral functioning. A next of kin (e.g., spouse) of each control subject was interviewed to corroborate his/her normal daily functioning.

All subjects or relative informants gave their written informed consent to the experimental procedure, which was approved by the Ethical Committee of San Raffaele Hospital.

### Story-based Empathy Task

ALS and HC were compared on the basis of the performance on the Story-based Empathy Task (SET), a non-verbal test targeting individuals’ ability to correctly attribute mental states to other agents [27, 36, 43]. Experimental procedure and stimuli were derived from tasks used in previous studies [44–46] and has been described for the first time in Cerami et al. [27]. Briefly, the SET includes two experimental conditions, namely the *Emotion Attribution* (EA) and *Intention Attribution* (IA) conditions, requiring to infer emotional and intentional states, respectively. The test also includes a control condition (*Causal Inference* or CI condition), involving the comprehension of physical cause-effect relationships.

Every condition includes six strips depicting different stories, and gives a maximum score of 6 for each condition. The task requires the interpretation of specific story-content for each strip. Firstly, three drawings describing consecutive moments of a story are presented in the upper half of the screen. So, the subject is asked to describe the story and to formulate a possible story ending. Then, three other drawings showing possible endings (i.e. plausible, implausible, and plausible but incorrect) are presented in random positions across different trials in the lower part of the screen. Finally, the subject is asked to select one of the three possible alternative endings (for further details and an example of the stimuli see [27]). EA condition includes strips based on the attribution of one of the six basic emotions (i.e., anger, fear, disgust, sadness, happiness, surprise). A failure to correctly select the story ending entails a misjudgment on intentions and emotions of the main character on EA and IA conditions, or an erroneous comprehension of causality on CI condition.

Group differences on the global score, as well as on single condition sub-scores, were analyzed with either parametric or nonparametric tests, depending on data distribution.

### Diffusion Tensor Imaging (DTI) data acquisition and analysis

All participants underwent Diffusion Tensor Imaging (DTI) on a 3-T Philips Achieva scanner (Philips Medical Systems, Best, NL) with an 8-channel head coil. Whole-brain DTI data were collected using a single-shot echo planar sequence (TR/TE = 8986/80 msec; FOV = 240 mm^2^; 56 sections; 2.5 mm isotropic resolution) with parallel imaging (SENSE factor, R = 2.5) and diffusion gradients applied along 32 non-collinear directions (b-value = 1000 sec/mm^2^). One non-diffusion weighted volume was also acquired.

Preprocessing and analysis of DTI data were performed via the FMRIB Software Library (FSL: http://fsl.fmrib.ox.ac.uk/fsl/fslwiki/‎) tools. Single-subject datasets were first corrected for eddy current distortions and motion artifacts, applying a full affine (linear) alignment of each volume to the no-diffusion weighting image. Corrected datasets were skull-stripped and, finally, as a result of the fitting of the diffusion tensor model at each voxel, maps of fractional anisotropy (FA) were generated. Whole-brain group analysis on FA was carried out via Tract-Based Spatial Statistics (TBSS), as described by Smith and colleagues [47]. We first computed a comparison between ALS and HC, and then we correlated whole-brain FA and EA scores in patient group. Voxelwise analyses were performed using Randomise–i.e., a software implemented in FSL performing a permutation-based nonparametric approach within the framework of the GLM–with 10,000 random permutations per contrast, and the Threshold-Free Cluster Enhancement as thresholding method [48]. The t-test comparison between patients and controls was considered significant at p<0.01 uncorrected. The threshold of significance relative to the correlation analysis between whole-brain FA and EA scores in ALS patients was set at p<0.05 corrected for multiple comparisons. The result maps were smoothed applying a Gaussian Kernel of 3 mm via the tbss_fill script. Localization of significant clusters was performed employing the JHU White-Matter Tractography Atlas and the JHU ICBM-DTI-82 White-Matter Labels [49].

Finally, we performed *off-line* analyses to explore the relationship between behavioral performances and mean FA values extracted from the significant results obtained in the whole-brain correlation analysis. Off-line correlations were computed with Statistica software (https://www.statsoft.com/), and p-values were corrected for multiple comparisons with the False Discovery Rate (FDR) method [50].

## Results

### SET impairments in non-demented sporadic ALS patients

The results from the whole group, of which these patients are a subsample, have been reported in Cerami et al. [35]. We compared ALS patients with HC on both SET global score and single condition scores. Since the SET scores did not fit a normal distribution (Liliefors test p>0.05), we used non-parametric statistics (Mann-Whitney U Test).

These analyses confirmed a significant failure in patients, compared with HC, in the overall attribution skills, measured with the SET global score (U = 40.50, p = 0.009, Cliff’s delta = 0.55). In order to ensure that this result was not driven by the presence of clinically evident cognitive or behavioral alterations, we repeated the same analysis excluding both *ALSci* and *ALSbi* subjects. Group comparison between *pure ALS* (n = 9) and HC on the SET global performances confirmed the presence of a significant decrease in attribution abilities in patients (U = 28, p = 0.018, Cliff’s delta = 0.55).

We then explored group differences in the EA, IA and CI conditions separately. This analysis confirmed that the emotion attribution condition was the only one impaired in patients (EA condition: U = 49, p = 0.028, Cliff’s delta = 0.46) (Table 2), with 6 out of 13 patients (2 ALSbi, 1 ALSci, 3 pure ALS) performing equal to or below the 5^th^ percentile of the controls’ scores distribution. See also S1 Fig for further details about ALS and HC performances.

**Table 2:** group differences on mental states attribution abilities.

	ALS (n = 13)	HC (n = 14)	U	p-value	Effect size
SET-GS	14.45, 16[9–17]	16.64, 17 [16–18]	40.5	0.009	0.55
SET-EA	4.63, 5 [2–6]	5.57, 6 [4–6]	49	0.028	0.46
SET-IA	5.09, 6 [3–6]	5.78, 6 [5–6]	65.5	0.135	0.28
SET-CI	4.72, 5 [3–6]	5.28, 5 [4–6]	80	0.565	0.12

The table shows mean, median and ranges of SET global (GS) score and sub-scores relative to single conditions (EA = emotion attribution; IA = intention attribution; CI = causal inference). Results of non-parametric comparisons (Mann-Whitney U Test) between patients (ALS) and healthy controls (HC), and the relative effect size (Cliff’s delta) are also reported.

### Microstructural correlates of emotion attribution impairment in ALS patients

**Fig 1:**
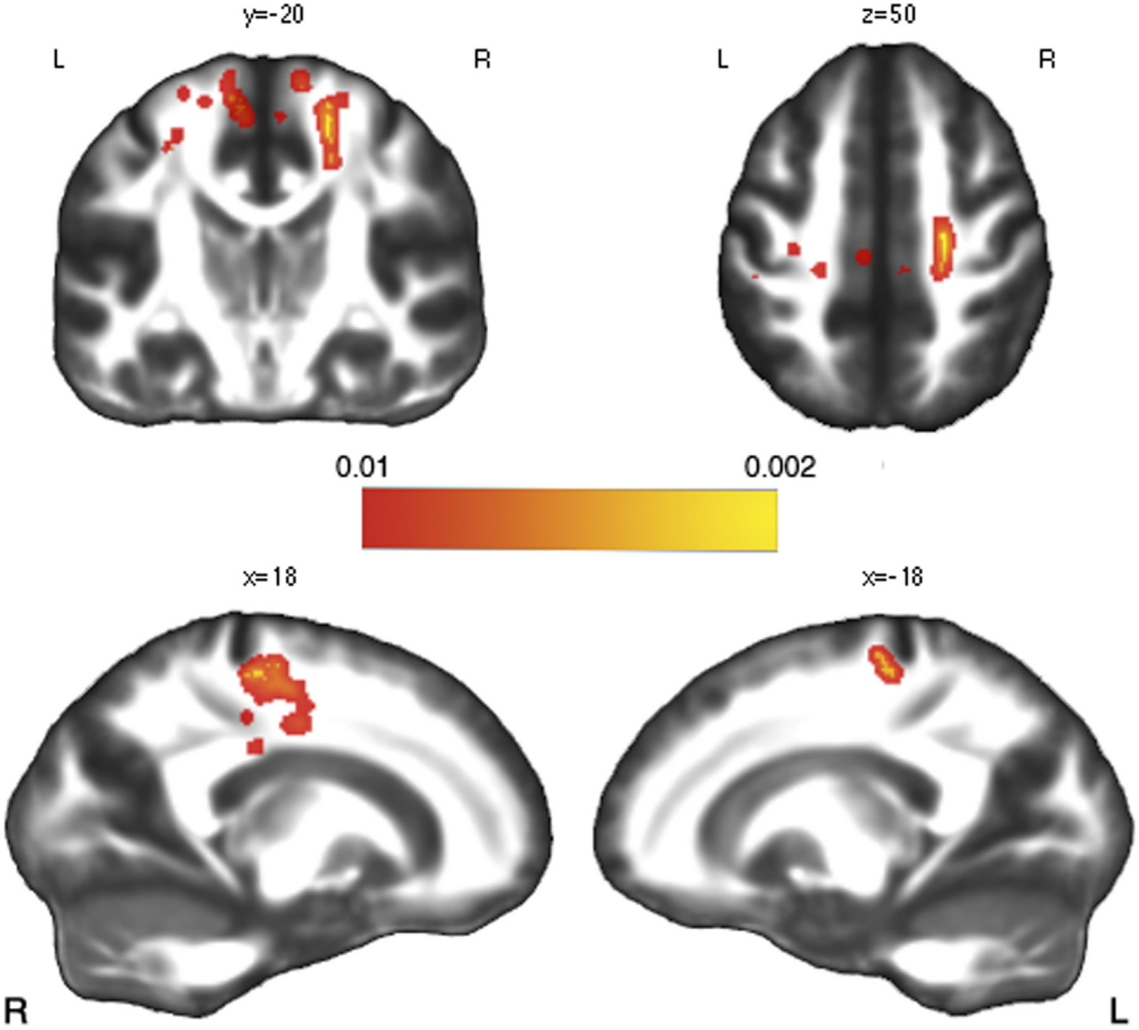
TBSS Whole-brain comparison between ALS patients and HC. Whole-brain comparison between ALS patients and HC showing a significant fractional anisotropy (FA) reduction (p>0.01 uncorrected) along the bilateral corticospinal tract (CST). Statistical map is superimposed to the FMRIB standard-space FA template.

Whole-brain voxelwise comparison on FA images revealed a significant reduction in ALS patients compared to HC involving both left and right corticospinal tract (Fig 1, p<0.01 uncorrected).

**Fig 2:**
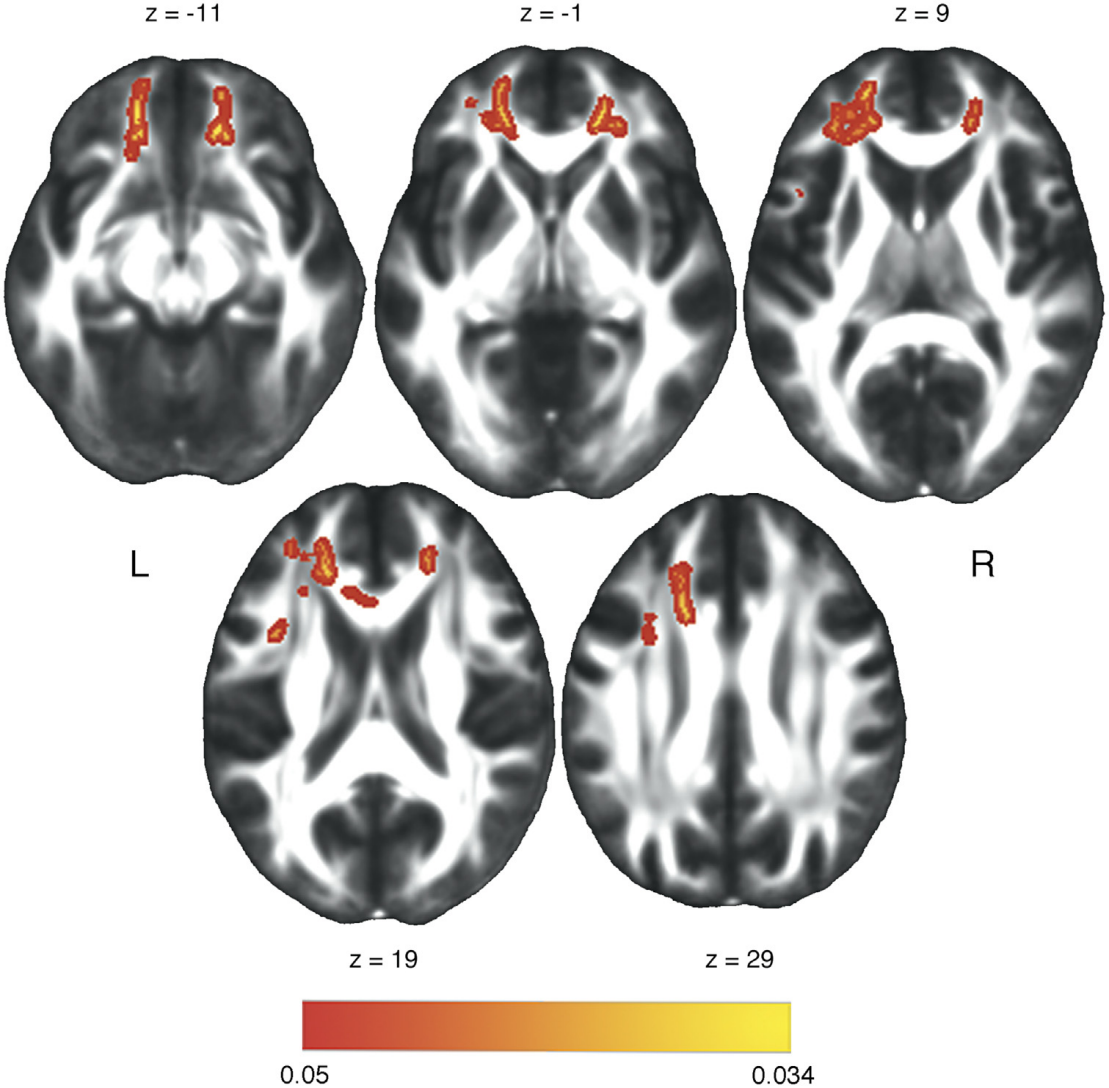
Correlation between white-matter microstructure and Emotion Attribution (EA) scores in ALS patients. Significant correlation between microstructural white-matter integrity (i.e., fractional anisotropy index) and EA performances in ALS patients. Statistical maps (p<0.05, FWE-corrected) are superimposed to the FMRIB standard-space FA template.

Whole-brain correlation analysis between skeletonized FA maps and EA scores in ALS patients highlighted a positive correlation (p<0.05 corrected for multiple comparisons) in four clusters localized along the fronto-temporal portions of ventral associative bundles, i.e., right inferior fronto-occipital (IFOF) and uncinate (UF) fasciculi, commissural fiber tracts (forceps minor and genu of the corpus callosum) and in the left superior longitudinal fasciculus (SLF) (Fig 2, Table 3). We then extracted mean FA values from binary maps of significant clusters from both patients and controls FA images. In these clusters, ALS patients showed a 1.8% reduction in diffusion coherence compared to HC, with 4/13 patients having mean FA values below the 5^th^ percentile of HCs’ distribution. We than computed a separate slopes model using FA from significant clusters as predictor of the EA scores. Results highlighted a main effect of group (F(1,23) = 8.86, p = 0.007), and a significant interaction effect between group and mean FA values of significant clusters (F(2, 23) = 14.22, p = 0.0001), with a strong positive correlation in ALS patients (r = 0.84, p = 0.0013) and no correlation in the control group (r = 0.01, p = 0.98).

**Table 3:** correlation between white-matter microstructure and Emotion Attribution scores in ALS patients.

clusters	localization	x,y,z	Cluster size	Partial correlation	p-value
#1	forceps minor	-18, 31, 19	1689	0.76	0.0002
#2	uncinate fasciculus, inferior fronto-occipital fasciculus-	15, 35, -11	570	0.79	0.000034
#3	superior longitudinal fasciculus	-32, 12, 24	77	0.65	0.006
#4	genu of corpus callosum, forceps minor	-5, 24 15	53	0.81	0.000011

The table shows localization, MNI coordinates (x,y,z) and size (number of voxels) of significant fractional anisotropy (FA) clusters emerged form the TBSS correlation analysis in ALS patients. Coefficients and p-values relative to the partial correlation analysis between EA scores and mean FA values extracted from significant clusters with disease duration (i.e., months from symptoms onset) as controlling variable are also reported. P-values were adjusted for multiple comparisons with the False Discovery Rate correction.

**Fig 3:**
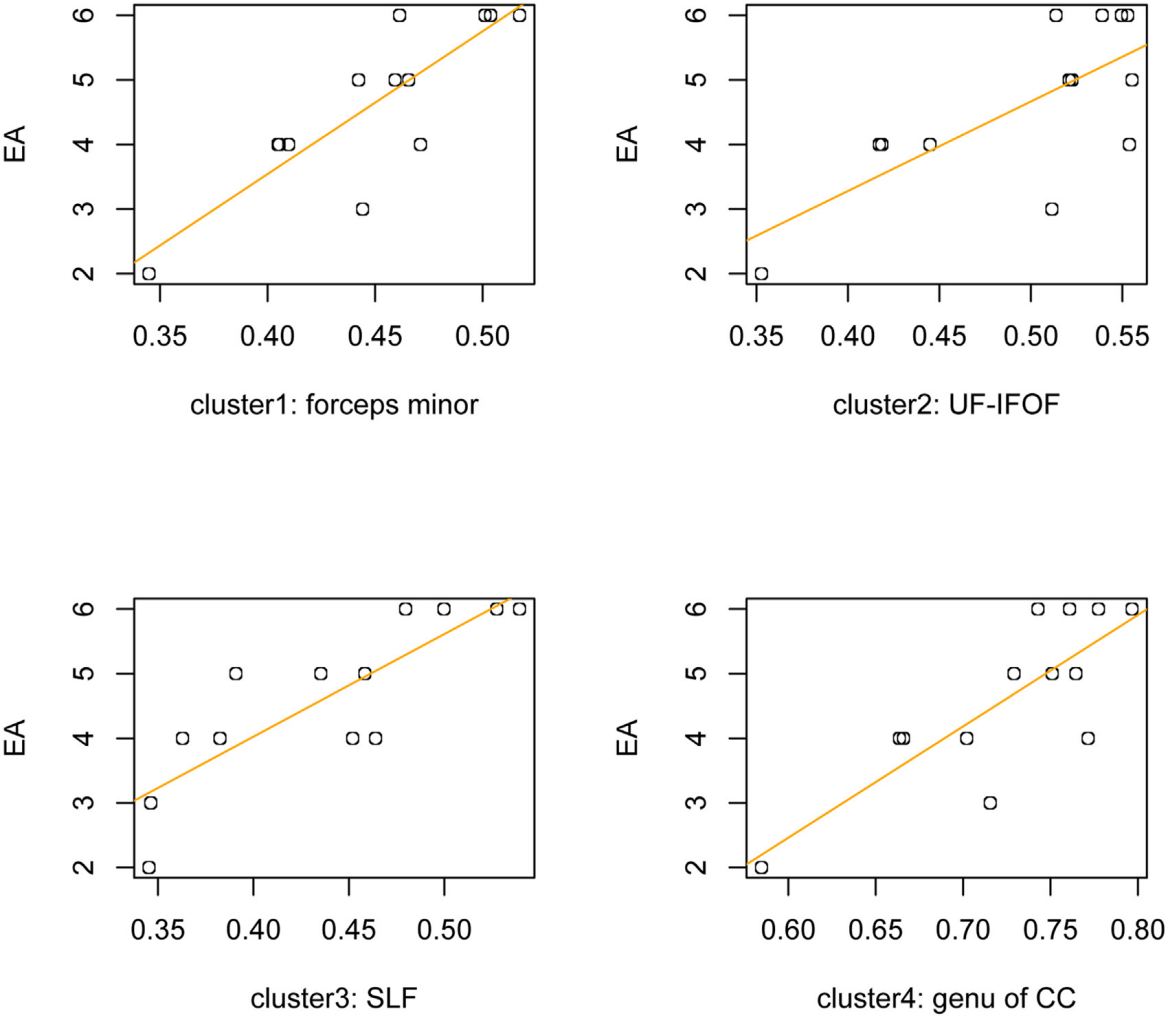
Correlation between white-matter microstructure and Emotion Attribution (EA) scores in ALS patients. The scatterplots illustrates significant correlations between EA scores and FA index within the significant clusters in ALS patients.

Finally, in order to exclude the possible impact of the high variability of disease duration on the relationship between emotion attribution skills and microstructural integrity in ALS patients (i.e., 25±22.37 months), we computed a partial correlation analysis between EA scores and mean FA values extracted from significant clusters, using the time interval (i.e., months) from symptoms onset as a controlling variable. The correlation coefficients remained significant (p<0.005, FDR corrected) even after controlling for disease duration (Fig 3, Table 3).

## Discussion

The present study explored for the first time the microstructural white-matter correlates of emotion attribution in a sample of non-demented sporadic ALS patients. Our results suggest that an impairment in empathic processing may occur in some cases, even in absence of deficits in other cognitive domains (i.e., *pure* ALS patients). Such an evidence is in contrast with the hypothesis that social cognition deficits in non-demented ALS patients are a consequence of executive impairment [51], and indicate that the failure in the ability to attribute mental states to another agent may be independent from the presence of additional extra-motor impairments. Notably, the overall attribution deficit emerged in ALS patients was driven by a specific alteration in emotional empathy (i.e., measured with the SET Emotional Attribution condition), suggesting a decline in the ability to identify emotional states in others.

Whole-brain voxelwise comparison on FA between ALS patients and healthy controls failed to find extra-motor microstructural changes on fronto-temporal pathways in our ALS sample, as previously reported [12]. Besides, the inclusion in this work of a smaller ALS patients sample without cognitive decline, a shorter disease duration and lower disease disability scores compared to previous literature [12, 52–53] may justify the lack of commissural and frontotemporal white-matter changes.

Conversely, the results of the whole-brain correlation analysis strongly support the notion of an extra-motor fronto-temporal and limbic involvement within the emotional empathy network in ALS. Pathological changes in the microstructural organization of intra- and interhemispheric fiber tracts conveying information between prefrontal, fronto-parietal and temporo-limbic areas may contribute to the decline of emotional empathy skills in ALS patients without cognitive decline in different ways. First, a lower diffusion coherence index along the right ventral bundles (IFOF/UF) may influence the processing of emotional information–i.e., from perception and modulation of the emotional characteristics of the visual percept (IFOF) to the visceromotor reaction to the stimulus (UF) [54–55]–by compromising the computations underlying the production of the embodied simulation that prompts the vicarious sharing of another’s feeling [31, 56]. Secondly, a decrease in microstructural integrity along anterior commissural connections (genu of corpus callosum and forceps minor) may interfere with the interhemispheric cooperation mediated by connections of the limbic system, which is crucial for the processing of affective valence of social stimuli. Indeed, coding, evaluation and interpretation of emotional cues within a social context, as well as inferences related to others’ mental states, require the integration of information and the coordination of the processing competences of the two hemispheres [57]. Finally, microstructural features of the SLF have been recently linked to interindividual differences in empathic concern, a measure reflecting emotional empathy [58]. The alteration of the fronto-parietal connectivity subserved by this bundle may thus affect the sensorimotor integration required to recognize and imitate other’s actions, a key mechanism for the development of social cognition abilities, included the expression of empathic responses [59].

Our results provide thus further evidence that white-matter impairment of frontal commissural fiber tracts (i.e., genu of corpus callosum and forceps minor), associative fronto-limbic (UF/IFOF) pathways is a distinctive characteristic of pathological conditions in which changes in the processing of the emotional valence of social stimuli represent a key cognitive feature [60–65]. In addition, in agreement with previous evidence showing a pathological disruption of rostral commissural and ventral bundles in ALS patients [12], we proved in our ALS sample the same pattern of white-matter changes that has been proposed as microstructural neuroanatomical marker of the progressive degeneration observed in bvFTD [66–68]. Notably, the white-matter bundles we found related to the impairment of empathic skills in ALS patients (i.e., genu of corpus callosum, IFOF, ILF) are those subserving the anatomical connectivity between prefrontal and limbic networks found to be altered in bvFTD resting state activity [39]. Together these findings further support the neurofunctional and neuroanatomical continuum between ALS and bvFTD.

It has been proposed that extra-motor impairments in ALS, including those affecting empathic abilities, might be the result of the involvement of mirror system networks (see [69]). In particular, such an impairment may be due to a breakdown in the automatic ‘resonance’ mechanism leading to a vicarious sharing of another’s feelings by coding a representation based on the bodily state of that agent in a given moment (i.e., *embodied simulation*) [56]. The neural computations supporting such a basic mechanism of emotional contagion involve both fronto-parietal (SLF) and fronto-limbic networks. The former component, associating action perception and production, modulates the activity of fronto-limbic regions through anatomical connection with the insular cortex. The latter (including the IFG, the ACC and the anterior insula) has been linked to interoceptive awareness and sense of subjectivity [70–71], and is engaged in mirror-like effects in both physical and social aversive contexts [72]. Accordingly, previous evidence reported macrostructural changes in core components of the emotional empathy network–namely, the fronto-insular cortex and the ACC–in ALS patients [27], in parallel with bvFTD [36], and the degree of the grey-matter damage highlighted in patients was positively associated to the severity of the impairment in the SET Emotional Attribution condition.

The main limitations of the study are the small sample size, and the lack of assessment of empathic behavior in real life situations. This work is, however, a proof-of-concept study aiming to provide preliminary and exploratory data. No previous data on the correlation between white matter damage and socio-emotional processing disorders in ALS have been so far reported. Although further studies are required to confirm the present findings, our results highlight important implications for clinical practice. The possible occurrence of socio-emotional impairments–and more generally of extra-motor symptoms–in early stages of ALS raises critical issues about patients’ advance directives and the need for effective supporting programs for patient and caregivers. A comprehensive assessment of different facets of non-motor (i.e., behavioral, cognitive and socio-emotional) deficits in ALS is needed to improve diagnostic accuracy and to support caregivers’ training and management strategies.

## Supporting Information

S1 Fig Participants’ performances related to mental attribution abilities. The figure describes the distribution of participants’ scores (see the color legend on the bottom) in the three SET conditions (y-axis: CI = causal inference, IA = intention attribution, EA = emotion attribution) in patients with amyotrophic lateral sclerosis (ALS) and healthy controls (HC). Values along the x-axis indicate the number of subjects in each group (13 patients and 14 controls).
(TIF)
