# Supplementary figures and images for: Microstructural Correlates of Emotional Attribution Impairment in Non-Demented Patients with Amyotrophic Lateral Sclerosis
Source: PLoS One. 2016 Aug 11;11(8):e0161034. doi: 10.1371/journal.pone.0161034 (PMC4981464; doi:10.1371/journal.pone.0161034)

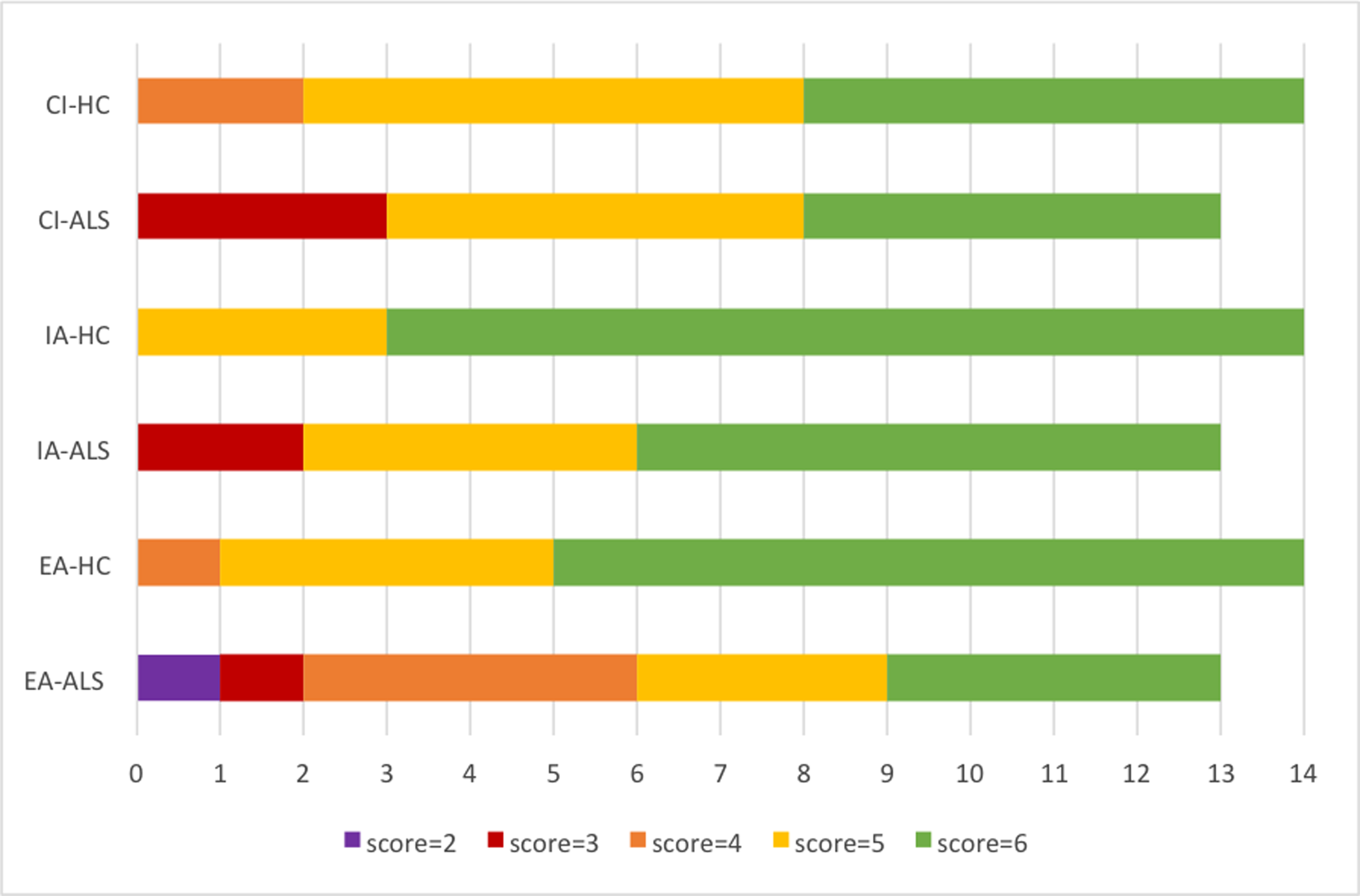

Supplement: S1 Fig — The figure describes the distribution of participants’ scores (see the color legend on the bottom) in the three SET conditions (y-axis: CI = causal inference, IA = intention attribution, EA = emotion attribution) in patients with amyotrophic lateral sclerosis (ALS) and healthy controls (HC). Values along the x-axis indicate the number of subjects in each group (13 patients and 14 controls). (TIF) [file pone.0161034.s001.tif]
